# Grand Challenges in Neuromodulatory Interventions

**DOI:** 10.3389/fpain.2021.700552

**Published:** 2021-07-02

**Authors:** Julie G. Pilitsis

**Affiliations:** Department of Neuroscience and Experimental Therapeutics and Professor of Neurosurgery, Albany Medical College, Albany, NY, United States

**Keywords:** chronic pain, neuromodulation, research, engineering, devices, spinal cord stimulation, TMS, deep brain stimulation

## The Frontiers Journal Platform

Frontiers encompasses over 80 journals across a range of academic disciplines including life sciences, engineering, health, and social sciences. The overarching mission focuses on accelerating scientific and technological innovation and societal progress. The initiative to develop a Frontiers in Pain Research stemmed from a recognition that pain affects more people than cancer, heart disease and diabetes combined (1). Under the leadership of Dr. Tony Yaksh, the organization has been launched to provide an open platform to disseminate research on pain. This pain platform is divided into sections on Neuropathic Pain, Cancer Pain, Pharmacological Treatment of Pain, Musculoskeletal Pain, Abdominal and Pelvic Pain, Headache, Pain Mechanisms, Pain Research Methods, Non-Pharmacological Treatment of Pain, Pediatric Pain, Geriatric Pain, Clinical Trialing and now, Neuromodulatory Interventions. Each of these sections function with their own Specialty Chief Editor to aggressively pursue the respective areas of pain research. I have accepted the Editorship for the specialty journal on Neuromodulatory Interventions. I am honored to serve in this capacity, as it offers a unique opportunity for me to further my interests in the collaborative advancement in regulating neuraxial processing by the application of medical and engineering sciences and technologies.

## What are “Neuromodulatory Interventions”?

Often when this question is asked, physicians and engineers first think of electrical devices, such as peripheral nerve spinal cord, ganglionic or deep brain stimulators (2). However, current technology has brought a myriad of technologies into the equation. Transcranial magnetic stimulation is at a state where its application can predictably alter neural function (3). Optogenetics presents unparalleled opportunities to excite or inhibit the activation profile of specific neural circuits (4). Focused ultrasound may be used to activate mechanically sensitive transfected brain structures or selectively open the local blood brain barrier (5) or alter the local delivery of therapeutics (6). Neuraxial and intra parenchymal delivery of therapeutics can acutely and chronically produce local changes in system function though focal therapeutic application (7, 8). In each case, I emphasize that the relevant subject matter includes the consideration of factors impacting upon the enablement of the intervention. Such work would include platform design as in catheters, leads, pumps, stimulatory systems and variable such as delivery and stimulation profiles.

Neurosurgical procedures for pain would not at face value be considered neuromodulatory, as they often involve ablating an area. However, interventions such as ventrolateral cordotomies have a long history of efficacy and utility in managing chronic pain in the appropriate patient. Cingulatomies can usefully result in changes in the affective aspects secondary to pain and severe stress (9). These interventions, aside from their efficacy in the management of chronic pain states, point to neuraxial targets where non-surgical approaches may be applied, as in regulating anterior cingulate function with optogenetics (10) or the neuraxial delivery of toxins, transfection platforms (viral and antisense) and novel stimulation paradigms leading to alterations in the function of specific circuits (11, 12). These advances, constituting proper subject matter for the journal, promise to give the neurosurgeon unparalleled opportunities to achieve minimally invasive interventions in specific neural pathways relevant to sensory, motor and other aspects of higher order processing.

For the purpose of this specialty journal, we will consider all of the above to be Neuromodulatory Interventions- that is not only the development and characterization of devices (stimulators, biological interfaces, pumps and catheters) but also other technologies, including biologics (as with optogenetics), which alter the functionality of specific neural systems.

As anticipated, a strong focus of this work will be on issues pertinent to pain processing. However, I believe to truly make an impact in this space and bring more effective therapies from bench to bedside, it is essential to leverage researchers' experience with neuromodulatory interventions, independent of the disease process, e.g., regulating aberrant electrical activity (seizures), or in the complex neural functions that underlie anxiety and emotional states, which aside from their impact upon daily function can severely impact upon the pain phenotype (as in PTSD) (13). In terms familiar to the researcher applying for funding in the United States, this means we are opting to take an NSF rather than an NIH approach. Specifically, we are focusing on the technologies instead of the disease process. Does that mean we will not consider observational studies and clinical trials on neuromodulation for pain? Absolutely not. We expect such research to be a considerable portion of our volume and have structured our editorial board accordingly.

## Eclectic Applications

Whether considering these neurosurgical interventions or neuromodulation, we are looking for research reports conducted in other diseases, when findings are relevant to expanding our understanding of the technologies and can be employed in the treatment of an array of diseases, including pain. The authors' cover letter and discussions in those reports should focus on how the insight they obtained in their submission is transferrable knowledge to the neuromodulation community as a whole. As pain is a tridimensional experience involving not only sensory processes but also cognitive and emotional processes, in many cases of brain stimulation, this extrapolation will be very straightforward. Pain can also develop from motor dysfunction, dysautonomia, and visceral dysfunction; thus, works tied to those topics in not only the brain, but also in the spinal cord and peripheral nervous system, are also valued.

## Role of Preclinical and Clinical Research

In addition to work across organ systems, we also realize the importance of research across species. In my own lab, we consider small animals, large animals and humans, as all may be employed to answer various questions. Rodent models are most often utilized to test new neuromodulatory techniques or to answer specific biological questions. Large animal models offer testing for devices and humans are the ones that may ultimately serve in the therapeutic translation. *In vitro* and *in situ* work will be considered when there is a direct correlation. Population studies are also solicited. Population based research reports may come from database studies and/or big data analysis related to those databases. These works often are powered to make meaningful assessments of real world experience with technologies. Further, they may offer guidance toward phenotyping which is difficult in the pain field, where symptoms and outcomes rely on self-reports from patients (14). Effective behavioral phenotyping is an essential component of future pain therapies.

## Definition of Biomarkers and their Role in Neuromodulation

Once phenotypes are established, the goal for any therapeutic becomes applying it to the right phenotype. Biomarkers that have been considered in the pain field to date have been based on functional imaging and / or physiological markers (14–16). Functional imaging generally has relied on fMRI or magnetoencephalography (MEG) techniques. Electrophysiological markers include those from the brain (EEG, LFPs, SUA) or from the spinal cord (ECAPs). While these markers offer insight into disease process, they may also provide value in “closing the loop” with neuromodulatory technologies. Closed loop systems enable patients to have the appropriate amount of therapy delivered based on their real-time needs. Given the tridimensional complexity of any given pain state, closed loop systems may be quite difficult to develop as they must account not only for the sensory components of pain but also the cognitive and emotional aspects.

While functional indices would most certainly involve electrophysiological measures, it is not difficult to envision that indices of neural activity might include feedback algorithms based on regional changes in brain blood flow or metabolism. These central indices may be merged with peripheral indices providing general insight into the activation state of the patient reflecting general emotional tone as defined by factors such as skin conductance, activity levels and/or heart rate variability. The current advances in measuring blood sugar or blood oxygenation reflect how such information can be gathered and integrated by wearable systems. Phenotyping, biomarkers and closed loop technologies are particular areas where cross-disciplinary collaboration is valued.

## Application and Implementation

Finally, we are not only considering technological innovations but also how that technology may arrive in the hands of the user, the clinician, and the consumer, the patient. Studies concerning socioeconomics are essential to therapy delivery. Reports focused on identification of barriers and strategies to overcome them are welcome. Often as healthcare technologies may actually increase healthcare disparities (17–19), it is important to address how these technologies will be designed, validated and implemented to serve those that historically have been underrepresented in medicine. Providing access to the latest advancements in healthcare is part and parcel to the open access nature of Frontiers. Though authors may at first consider this a barrier to submission, providing information to all researchers and clinicians should be a fundamental tenet of science and of medicine to advance global knowledge. Researchers and clinicians in countries with less resources often have fees waived or heavily discounted for submissions. In higher resourced countries, institutions may also partner with Frontiers for standing discounts. Visit https://www.frontiersin.org/about/institutional-membership to determine if your institution partners with Frontiers.

Taken together, in conjunction with Dr. Tony Yaksh, I am thrilled to announce Frontiers in Pain Research: Neuromodulatory Interventions. We welcome your submissions and after 30 publications, all contributions will be retrospectively and then prospectively indexed in PubMed. We aim to reach that goal within 1 year from today. We strongly feel that cross-pollination of ideas is essential for the future of neuromodulation and we look forward to your partnership as an author and potentially as a reviewer. Please reach out to me personally with any ideas— jpilitsis@yahoo.com.

“If you want to go fast, go alone. If you want to go far, go together.” - Old African Proverb.

## Author Contributions

JP wrote the first and final draft of the manuscript and approved the submitted version. It was reviewed by TY.

## Conflict of Interest

JP is a consultant for Boston Scientific, Nevro, Medtronic, Saluda and Abbott and receives grant support from Medtronic, Boston Scientific, Abbott, Nevro, NIH 2R01CA166379-06 and NIH U44NS115111. She is the medical advisor for Aim Medical Robotics and Karuna and has stock equity.
